# Open airway surgery for post-COVID laryngotracheal stenosis

**DOI:** 10.1007/s00405-024-08533-z

**Published:** 2024-02-26

**Authors:** Lluís Nisa, Hajdi Leroyer, Kishore Sandu

**Affiliations:** Department of Otorhinolaryngology, Head and Neck Surgery, Lausanne University Hospital (CHUV), Lausanne, Switzerland

**Keywords:** COVID, Laryngotracheal stenosis, Airway stenosis

## Abstract

**Introduction:**

This study reports our experience with open reconstructive surgery in patients with laryngotracheal stenosis (LTS) following prolonged intubation and/or tracheostomy in the context of COVID-19.

**Methods:**

All patients underwent a preoperative endoscopic airway assessment. Posterior glottic lesions were graded according to the Bogdasarian classification, subglottic-tracheal lesions according to the Cotton-Myers classification and postoperative complications reported by the Clavien–Dindo classification. We report postoperative outcomes and functional results in this patient subset.

**Results:**

We include 14 patients diagnosed to have post COVID LTS, one female and 13 males. This group was compared with a control group, diagnosed with LTS following prolonged intubation. In the COVID group, mean age of patients at the time of the airway surgery was 52.1 ± 16.8 years (range: 13.7–76.3). More than half the patients were multi-morbid with hypertension and obesity being the most common conditions. Eleven patients had multi-site stenoses. Open surgical interventions performed were tracheal resection and anastomosis, laryngotracheal reconstruction and extended cricotracheal resection, and postoperative complications were seen in 12 (85.6%) patients. 70% patients with pre-existing tracheostomy were decannulated. Oral swallowing was not tolerated in one-fifth of the patients and a significant number of them have poor voice quality.

**Conclusion:**

Post-COVID pandemic, airway surgeons are seeing an increased number of patients with complex LTS, and we report significant postoperative complications in this patient subset. Decannulation rates, voice and swallowing results are poor in patients with glottic involvement as compared to isolated tracheal stenosis.

## Introduction

**Fig. 1:**
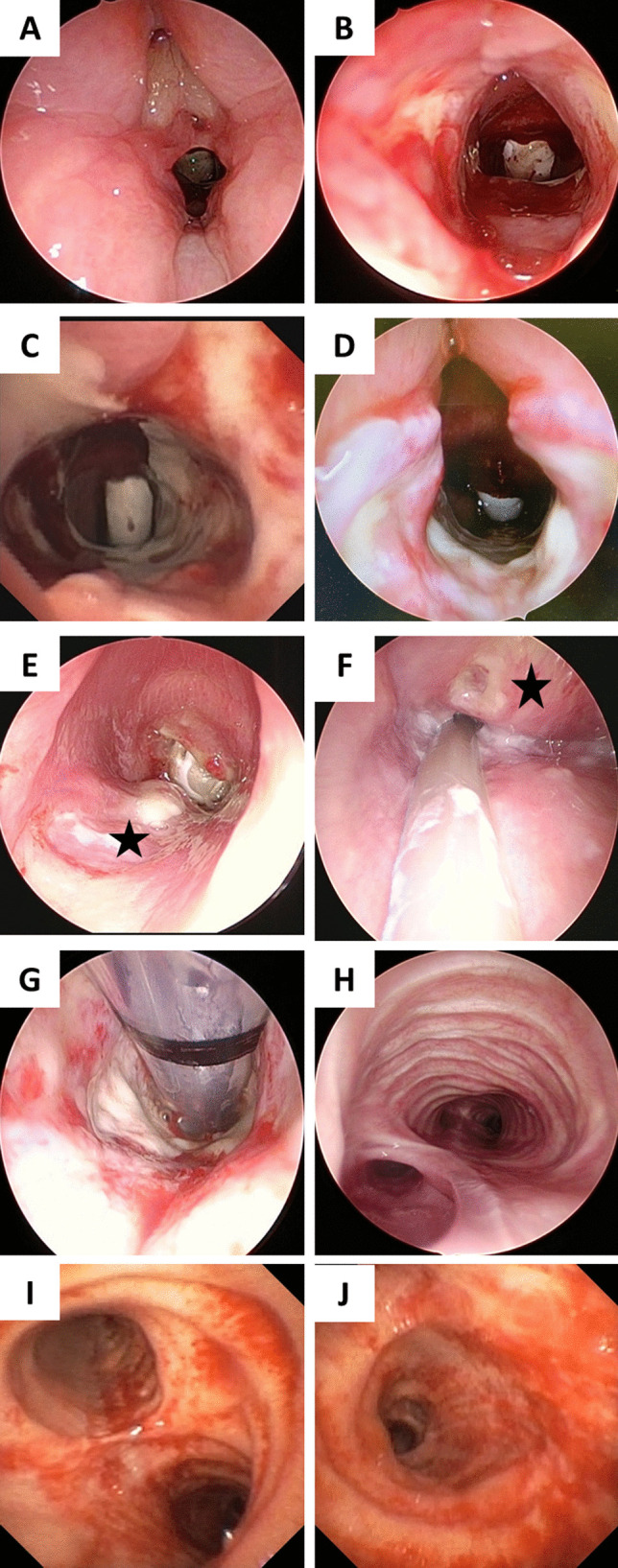
COVID-related endotracheal tube intubation lesions. **A** Inter-arytenoid granulations; **B** Cicatricial furrows, where there is endotracheal tube decubitus and mucosal denudation from the medial aspect of both arytenoid cartilages; **C** Circumferential denudation of subglottic mucosa; **D** Complete absence of the posterior glottic mucosa and exposure of both cricoarytenoid joints; **E** Pharyngolaryngeal fistula (*black colored star* corresponds to the endocricoid lesion); and **F** retro-cricoid lesion in the same patient (*black colored star*); **G** endotracheal tube cuff lesions in the upper trachea; **H** Large tracheoesophageal fistula; **I**, **J** Severe tracheobronchial inflammation

The laryngotracheal stenosis committee of the European Laryngological Society published a report [1] following the first wave of the COVID-19 pandemic, alerting of the possibility of a surge in the number of airway injuries secondary to prolonged intubation and tracheostomy. The various airway lesions seen in COVID patients (Fig. 1) due to *prolonged intubation *(*PI*) are [2] vocal cord(s) edema/granuloma, vocal cord(s) immobility—either due to recurrent laryngeal nerve palsy or ankylosis of one- or both cricoarytenoid joints, posterior glottic stenosis (PGS), vocal cords synechiae, subglottic retention cysts/ulcers/and stenosis. The endotracheal tube (ETT) occupies the posterior glottis and the subglottis and following a PI may denude the airway mucosa and progressively develop PGS.

Airway lesions following a *tracheostomy* are tracheal stenosis, suprastomal collapse and an A-shape deformity that typically manifests several weeks after decannulation and dynamically obstructs the airway.

Airway pathology seen in *both* PI and tracheostomy are cuff and tip of the tube/cannula stenotic lesions and tracheoesophageal fistula.

More complicated LTS with serious functional consequences can occur following a *dual insult* of combined airway trauma caused by PI and tracheostomy and the presence of severe viral inflammation [2–4]. The goal of this study is to report our experience with open reconstructive laryngotracheal surgery in a cohort of patients with COVID-related LTS.

## Methods

### Cohort, inclusion criteria and data extraction

For this study, an institutional board review approval and local ethics committee authorization was obtained (VD CER 2020-01500), and was conducted at a quaternary airway care center. During this period, both local and referred patients (from the rest of Switzerland and abroad) were treated. Patients from external institutions were referred for airway correction by other ENT surgeons, thoracic-general surgeons, and sometimes intensivists. Potential patients for inclusion were identified through the prospectively documented database of patients undergoing open airway management of LTS, and excluding those who were cured following endoscopic treatment. We included patients with LTS resulting from COVID-related intubation, without age limitation, with a full pretreatment workup consisting of endoscopy and other exams when required. We compared these patients with a control group that consisted of patients with benign LTS following prolonged intubation or post-tracheostomy. The control group excluded patients with idiopathic SGS and Wegner’s granulomatosis. The data collection for the COVID group was from July 2020 up to October 2022, and the control group included patients operated 24 months prior to the pandemic.

In both groups, the data collected included gender, age, presence of comorbidities, reason and length of intubation, presence of tracheostomy, detailed endoscopic findings, type of surgery, complications and their management, outcome and follow-up data. Majority of patients were referred from other university hospitals in Switzerland and abroad, and relevant data was electronically transferred to our unit. All patients had at least one endoscopy at our institution prior to their discharge.

In COVID patients who were referred to our unit, following information was missing in the data collected: intubation circumstances (in an emergent situation or planned), difficult intubation assessment details, experience of the person intubating, and endotracheal tube/tracheostomy cannula sizes. No prior endoscopic treatment was performed to treat neither early intubation lesions nor the acquired airway stenosis.

Surgical complications were classified according to the Clavien–Dindo classification [5].

Recorded data were double-checked in a non-blinded manner by two authors (LN and KS). In case of discordance, agreement was reached through discussion. Data are presented as summary or descriptive statistics.

### Endoscopic airway evaluation

All included patients underwent a complete endoscopic workup prior to any treatment. This consisted of a flexible awake trans-nasal laryngo-tracheo-bronchoscopy to evaluate laryngeal dynamics and particularly vocal cord movements, the trachea and the bronchi. Prior to passing the flexible scope distally, the vocal cords, subglottis and the trachea were sprayed with 1% Oxybuprocaine (Novesine-CHUV, Switzerland). In patients having a tracheostomy and an optimal glotto-subglottis, the cannula was removed and the stoma was covered with a sterile humid gauze and dynamics of the stoma and the distal airway were recorded. The anesthesia plane was subsequently deepened to perform direct pharyngo-laryngoscopy and tracheo-bronchoscopy, suspension microlaryngoscopy (SML) and esophagoscopy. The arytenoid cartilage(s) was passively palpated by a blunt probe for cricoarytenoid fixation. Under SML, the vocal cords were spread apart and the posterior glottis—subglottis was examined using angled endoscopes and palpated with a blunt probe for deficient posterior cricoid plate. Bronchoalveolar lavage was performed and the aspirate was sent for microbiology examination and antibiotic sensitivity. An esogastroscopy was performed routinely in all patients.

### Surgical treatment

The various open surgical techniques have been previously described [6]. Segmental tracheal resection and anastomosis (TRA) was done for an isolated tracheal pathology, laryngotracheal reconstruction LTR (full laryngofissure, posterior cricoid split and rib cartilage graft expansion) was done for posterior glottic ± minor subglottic stenosis and an extended cricotracheal resection and anastomosis (E-PCTR) was performed for severe glotto-subglottic stenosis.

### Follow-up and outcomes

The majority of patients in both groups (COVID and control) are being followed up outside of our institution, have at least 24 months of follow up and the most current information is shared by the referring doctors by way of electronic mails.

The main outcome variable of interest concerned the airway, particularly in terms of tracheostomy cannula removal. Other outcome variables included swallowing and voice outcomes. The later were evaluated subjectively as per the following criteria: (a) normal voice, (b) mild dysphonia (hoarse voice with some difficulties to hear or understand in a noisy environment), (c) moderate dysphonia (weak voice or ventricular band phonation with easy fatigability), and (d) severe dysphonia (breathy voice with difficulty to communicate).

No instrumental voice assessment was performed pre-operatively.

## Results

In the COVID group, 14 patients fulfilled the inclusion criteria, and included 13 males and one female. Of these patients, one patient was treated for COVID-related disease at our institution, 5 were referred from other university hospitals within our country and eight were from abroad. Their mean age at the time of open reconstructive surgery was 52.1 years (range 13.77 to 76.31). All these patients except one had comorbidities and over half of them were multimorbid, with obesity and hypertension as main pathologies (Table 1). The median BMI in obese patients (*n* = 7) was 33 kg/m^2^. Three quarters of the patients were intubated because of COVID-related acute respiratory distress syndrome (ARDS), and the remaining quarter because of progressive dyspnea without a formal diagnosis of ARDS. The mean length of intubation was 17 days (range 7 to 28). Three quarters of all patients received tracheostomies prior to open airway reconstruction.

**Table 1:** Patient charateristics

Characteristic	Covid group (*n* = 14)		Control group (*n* = 23)	
	*N*	%	*N*	%
Mean age at surgery (y, range)	52.1 ± 16.81 (13.77–76.31)		52.7 ± 12.2 (30.0–77.6)	
Gender				
Female	1	7.69	20	86.96
Male	13	92.31	3	13.04
Comorbidities				
None	1	7.69	8	34.78
Multiple	8	53.85	10	43.48
Hypertension	8	61.54	7	30.43
Obesity	7	53.85	4	17.39
Diabetes type 2	3	23.08	3	13.04
COPD	3	23.08	0	0
Peripheral vascular disease	2	15.38	0	0
Epilepsy	1	7.69	2	8.69
Stroke	1	7.69	3	13.04
Sofferman syndrome	1	7.69	0	0
Reason for intubation				
Respiratory	14	100	3	13.04
Neurologic	0	0	7	30.43
Tracheostomy prior to reconstruction				
Yes	10	76.92	12	52.17
No	4	23.08	11	47.83
Mean length of intubation (days)	17 ± 7 (7–28)		N/A	
Interval between extubation and the first diagnostic endoscopy (months)	3.89 ± 3.54 (0.33–10.80)		N/A	

*y* years, *COPD* chronic obstructive pulmonary disease, *N/A* not available

The control group with 23 patients (20 females, 3 males) had prior intubation either due to respiratory or neurologic causes and 12 (52%) patients had tracheostomy prior to their referral to our unit.

**Fig. 2:**
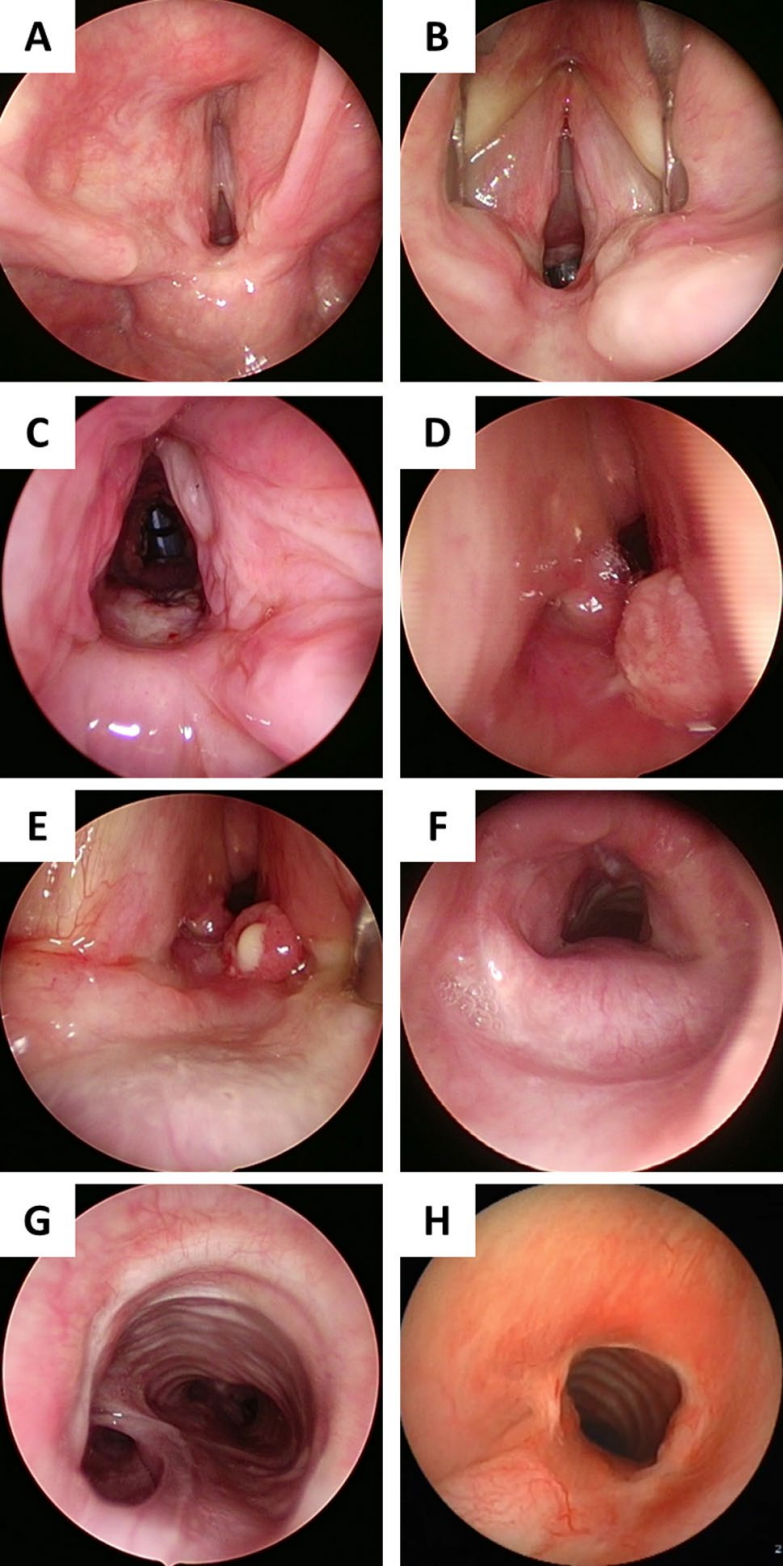
Case series of patients with complex airway stenosis. *Patient 1*: **A** pre-operative endoscopic view showing bilateral cricoarytenoid joint ankylosis; **B** view showing the glottic status with a vocal cord spreader in place. Note the limited inter-arytenoid distance; **C** post-operative view after posterior cartilage graft expansion. The patient was successfully decannulated. *Patient 2*: **D**, **E** pre-operative view of grade III subglottic stenosis, and right vocal cord immobility due to cricoarytenoid joint fixation; **F** endoscopic view following a two-stage extended cricotracheal resection and anastomosis. The patient was successfully decannulated. *Patient 3*: **G** pre-operative view of a large (5 cm) tracheoesophageal fistula TOF; **H** endoscopic view after a single stage tracheal resection and anastomosis and closure of the TOF

In the COVID group, the interval between extubation, initial tracheostomy and diagnostic endoscopy was 3.89 months (range 0.33 to 10.80). In terms of initial endoscopic findings, most patients (76.9%) had multilevel stenoses. The sites of stenosis were posterior glottis and subglottis (*n* = 7) and trachea (*n* = 6), one patient had a large (5 cm) tracheoesophageal fistula (Table 2). In the control group, posterior glottic and isolated tracheal stenosis were seen in 4 patients each, and glotto-subglottic stenosis was present in 15 patients. Table 2 summarizes the stenosis grades. Patients with tracheal stenosis in both groups had lumen obstruction of at least 70% (Fig. 2, Table 2).

**Table 2:** Endoscopy and surgery details

Endoscopic findings	Covid (nb, percentage)	Control group (nb, percentage)
PGS	*n* = 4; 28.5% Bogdasarian Type 3: 1 Type 4: 3	*n* = 4; 17.4% Bogdasarian Type 3: 3 Type 4: 1
G-SGS	*n* = 3; 21.4% SGS Cotton-Myer Grade II: 1 Grade III: 2	*n* = 15; 65.2% SGS Cotton-Myer Grade I: 2 Grade II: 3 Grade III: 7 Grade IV: 3
TS	*n* = 6; 42.8% Grade III: 3 Grade IV: 1 A frame deformity: 2	*n* = 4; 17.4% Grade III: 2 A frame: 2
TOF	*n* = 1; 7.1%	*n* = 0

*PGS* posterior glottic stenosis, *G-SGS* glotto-subglottic stenosis, *TS* tracheal stenosis, *TOF* tracheo-oesophageal fistula

In the COVID group, 7 patients received TRA, 4 had LTR, and 3 patients had E-PCTR. All 7 patients with isolated tracheal lesions had single stage operations. One patient with severe tracheal stenosis with no tracheostomy was sent to our hospital with an indwelling silicone tracheal stent placed for 2 years that was removed 2 weeks prior to the TRA. In E-PCTR and TRA, an average of 4 tracheal rings were removed (range 3 to 6). One case required a sternotomy to mobilize the trachea due to extensive peristomal adhesions (Table 3).

**Table 3:** Type of surgery

Type of surgery	Covid group		Control group	
	*N*	%	*N*	%
LTR	4	28.5	3	13
PCTR	0	0	8	34.8
EPCTR	3	21.4	5	21.7
TRA	7 (1 was for TOF repair)	50.0	7	30.5
Number of tracheal rings removed, mean (range)	4 (1–6)		3 (1–6)	
LT-Mold insertion	7	53.9	9	39.1
Sternotomy	1	7.6	0	0

*LTR* laryngotracheal resection, *PCTR* partial cricotracheal resection, *EPCTR* extended partial cricotracheal resection, *TRA* Tracheal resection and anatomosis, *TOF* tracheo-oesophageal fistula

Histological examination of resection specimens (EPCTR, TRA) in the COVID group revealed altered inflammatory neutrophils, lymphocytes and plasma cells, as well as presence of micro-abscesses responsible for chondrolysis.

Median number of endoscopies in the COVID group to achieve an optimal airway were 6 (IQR: 2–15). Endoscopic interventions after open reconstructions included balloon dilation (12 patients) and CO_2_ laser arytenoidectomy-posterior cordotomy (4 patients) to achieve an optimal airway.

In the COVID group, the majority of patients had complications (12/14; 85.7%) and 2 patients required an additional open revision surgery. In the control group, complications were seen in 7/23 (30.4%) patients, and none had a revision surgery. The distribution of these complications according to the Clavien–Dindo classification is detailed in Table 4.

**Table 4:** Surgical complications and revision surgery

Feature	Covid group	Control group
Complications		
Absent	2 (14.3%)	16 (69.5%)
Present	12 (85.7%)	7 (30.4%)
Clavien–Dindo grade II		
Tracheostomy/peristomal infection	1	0
Mediastinitis	1	0
Pulmonary embolism	1	0
Clavien–Dindo grade IIIa		
Neck abscess	1	0
Persistent tracheo-cutaneous fistula	1	3
Graft necrosis or exposure	1	1
Bronchoaspiration	0	1
Clavien–Dindo grade IIIb		
Anastomotic dehisence and TOF	1	0
Early stenotic recurrence	3	0
Clavien–Dindo grade IVa		
Nonfunctional larynx with massive aspiration + recurrence of stenosis	1	1
Early post-operative emphysema with respiratory compromise needing intubation	0	1
Endoscopic interventions following the airway surgery to achieve an optimal airway		
Dilatation	12	8
Laser ablation (arytenoidectomy/posterior cordectomy)	4	7
Re-insertion of laryngeal prosthesis	1	0
Revision surgery		
Single stage tracheal resection-anastomosis	1	0
LTR	1	0

Seven out of ten patients (70%) in the COVID group with prior tracheostomy were decannulated within a mean duration of 2.5 months. One patient continues to have severe aspiration and grade III recurrence of stenosis with no possibility of any salvage surgery and awaits a total laryngectomy. One patient died due to non-airway related causes. In the control group, all patients with tracheostomy were successfully decannulated. In both groups, there was no significant difference in the time required to achieve decannulation (Table 5).

**Table 5:** Outcomes

Characteristic	Covid group		Control group	
Time follow-up [months, mean ± SD (range)]	5.11 ± 4.45 (0.85–14.36)		5.65 ± 6.19 (0.56–25.72)	
Time till decannulation [months, mean ± SD (range)]	2.5 ± 0.62 (1.77–3.29)		2.21 ± 1.46 (0–5.29)	
Preoperative swallow				
Oral	12	84.62	22	95.65
NGT/PEG	1	7.69	1	4.35
Combined	0	0.00	0	0.00
Postoperative swallow				
Oral	11	76.92	23	100
NGT/PEG	1	7.69	0	0.00
Combined	2	15.38	0	0.00
Dysphonia				
None	4	23.08	13	56.52
Moderate	8	61.54	6	26.09
Severe	2	15.38	4	17.39

Currently, in the COVID group, 3 (21.4%) patients cannot tolerate oral feeds and are fed by a gastrostomy tube. Ten (71.4%) patients continue to have moderate-severe dysphonia. In the control group, all patients tolerate oral feeds and 10/23 patients (43.5%) have significant voice complaints.

## Discussion

In this study we report our experience with open airway reconstruction in patients with LTS following COVID related prolonged intubation ± tracheostomy, and our main findings in these patients are: (1) most patients had comorbidities, particularly obesity and hypertension; (2) LTS was multilevel; (3) following open airway surgery, there was a high rate of complications and approximately one-third of the patients required revision surgery; (4) the rate of decannulation was of 70%; and (5) functional outcomes in terms of swallowing and voice were poor when the glottis was preoperatively involved.

Endotracheal intubation and tracheostomy are well known causes of laryngotracheal stenosis (LTS). The degree and depth of injury mainly depends on duration of intubation, size of the tracheal tube, depth of sedation, patient’s general conditions (cardiovascular diseases, diabetes, and obesity playing an ominous role), and superimposing local infections. Due to lack of prospective studies comparing open and dilatational tracheostomy, it is difficult to say which amongst the two techniques cause more airway damage. Nevertheless, it is thought that dilatational forces may cause fractures of the tracheal cartilages and predispose to stenosis [7].

Several works have now identified mechanisms [3] specifically related to COVID-19 treatment, which increase the susceptibility of the larynx and the trachea to intubation and tracheostomy complications. These are: (1) the pronation maneuvers wherein position of an intubated patient is alternated between being dorsal decubitus to ventral decubitus to improve lung ventilation in case of an adult respiratory distress syndrome, (2) prothrombotic and antifibrinolytic state of the patients affecting the laryngo-tracheal and esophageal microcirculation—thus predisposing the mucosa to more ischemia and necrosis, (3) high viral replication in the tracheal epithelium could weaken the mucosa, (4) chronic high dose corticosteroid use thins down the tracheal mucosa, (5) lower PaO_2_/FiO_2_ ratio causes increased hypoxia of the laryngo-tracheal mucosa, (6) emotional and physical exhaustion of the care givers can add to the iatrogenic trauma, (7) existing comorbidities. The posterior glottis and subglottis endure most of the trauma and the injuries could evolve into uni- or bilateral cricoarytenoid fixation.

Type 2 diabetes mellitus is a well-known predisposing factor for LTS, being probably related to different metabolism and fibroblast populations [8]. In overweight patients, the optimal ventilatory pressure is set at higher levels due to their lower thoracic compliance, and this can contribute to the ischemic mechanism leading to post-intubation LTS formation.

To the best of our knowledge, this report describes the largest experience from a single center on open airway surgery for complex LTS following prolonged intubation and tracheostomy for COVID infection. Below are important findings we encountered while treating these patients:

- 1. Characteristics of patients and site of stenosis
  Hypertension and obesity were the main comorbidities in our series, though many had multiple comorbidities. Various predictors of difficult laryngeal exposure [9] in our patients prior to the intubation were not known, though in our hands we report that the pre- and postoperative endoscopy was challenging. In patients with glotto-subglottic lesions, we preferred using the rigid bronchoscope to ventilate and also to optimize the airway after the stenosis-corrective surgery using laser and balloon dilation [2]. In these patients, the vents on the rigid bronchoscope remained outside the glottis and were blocked using Steri strips to optimize ventilation pressures. The vent blocking was not required when optimizing the distal airway.
- 2. Post operative complications, decannulation and functional results
  Prolonged endotracheal intubation leads to mucosal damage and inflammation, granulation tissue formation, perichondritis, chondritis and subsequent cicatricial stenotic tissue formation. In our experience, the *double insult* of prolonged intubation trauma with large ETTs and the severe COVID-19 infection predisposed to cause intense inflammatory response and led to more serious LTS. The damage in the posterior commissure due to the ETT in the airway and a large size nasogastric tube combined with frequent positional changes was severe to cause chondrolysis of the posterior cricoid cartilage. Additional chronic steroid therapy for ARDS thinned the pharyngo-laryngeal mucosa and could be breached during a cricoid expansion surgery complicating with a pharyngolaryngeal fistula (PLF) formation as was seen in one of our patients. This patient further received an extended cricotracheal resection and closure of the PLF using a vascularized tracheal flap.
  In the COVID group, patients undergoing treatment for an *isolated tracheal lesion* had excellent postoperative results—early decannulation, no complications and better functional results, as was also observed by other colleagues [4, 10]. However, patients with *glottic involvement* presented with more severe form of posterior glottic stenosis, multi-level stenoses and relatively more scarred glottis, causing poor decannulation rates, and voice and swallowing results as compared to patients in the control group.

There are several drawbacks in this report, the first being its retrospective design and limited patient number. Because of the referral pattern of our patients, several important aspects related to the intubation, tracheostomy, and prior endoscopic treatment that can influence the outcomes were missing. While comparing the two groups, one can argue that more patients in the non-COVID group received PCTR and none in the COVID group. We did this because complications are somewhat identical and comparable in all patients undergoing an airway resection and anastomosis (PCTR or EPCTR or TRA).

In our experience, after the COVID pandemic large number of patients can still be recuperating in rehabilitation centers, and there could be a significant delay prior to the diagnosis of their airway stenosis and starting the treatment. Therefore, experience from multiple centers with larger number of patients will give clear treatment guidelines in treating these difficult patients who might be increasingly seen in the coming years.

## Conclusion

Post COVID pandemic, airway surgeons are seeing an increased number of patients with significant laryngotracheal stenosis following prolonged intubation and/ or tracheostomy, and we report significant postoperative complications in this patient subset. Decannulation rates, voice and swallowing results are poor in patients with glottic involvement as compared to isolated tracheal stenosis.

## References

[1] Piazza C, Filauro M, Dikkers FG (2021). Long-term intubation and high rate of tracheostomy in COVID-19 patients might determine an unprecedented increase of airway stenoses: a call to action from the European Laryngological Society. Eur Arch Otorhinolaryngol.

[2] Sandu K (2021). Laryngotracheal complications in intubated COVID-19 patients. Clin Med Insights Case Rep.

[3] Fiacchini G, Tricò D, Ribechini A (2021). Evaluation of the incidence and potential mechanisms of tracheal complications in patients with COVID-19. JAMA Otolaryngol Head Neck Surg.

[4] Onorati I, Bonnet N, Radu DM, Freynet O, Guiraudet P, Kambouchner M, Uzunhan Y, Zogheib E, Martinod E (2022). Laryngotracheal post-intubation/tracheostomy stenosis in COVID-19 patients. Front Surg.

[5] Dindo D, Demartines N, Clavien PA (2004). Classification of surgical complications: a new proposal with evaluation in a cohort of 6336 patients and results of a survey. Ann Surg.

[6] Monnier P (2011). Pediatric airway surgery. Management of laryngotracheal stenosis in infants and children.

[7] Pancani S, Virga A, Spina R, Peris A, Corvi A (2018). Experimental measurement of forces during percutaneous dilatational tracheostomy. Proc Inst Mech Eng Part H J Eng Med.

[8] Lina IA, Berges A, Ospino R (2022). Identifying phenotypically dis tinct fibroblast subsets in type 2 diabetes-associated iatrogenic laryn gotracheal stenosis. Otolaryngol Head Neck Surg.

[9] Pinar E, Calli C, Oncel S, Selek B, Tatar B (2009). Preoperative clinical prediction of difficult laryngeal exposure in suspension laryngoscopy. Eur Arch Otorhinolaryngol.

[10] Piazza C, Lancini D, Filauro M, Sampieri C, Bosio P, Zigliani G, Ioppi A, Vallin A, Deganello A, Peretti G (2022). Post-COVID-19 airway stenosis treated by tracheal resection and anastomosis: a bicentric experience. Acta Otorhinolaryngol Italica.

